# Bayesian model-based inference of transcription factor activity

**DOI:** 10.1186/1471-2105-8-S2-S2

**Published:** 2007-05-03

**Authors:** Simon Rogers, Raya Khanin, Mark Girolami

**Affiliations:** 1 Bioinformatics Research Centre, Department of Computing Science, University of Glasgow, Glasgow, UK; 2 Department of Statistics, University of Glasgow, Glasgow, UK

## Abstract

**Background:**

In many approaches to the inference and modeling of regulatory interactions using microarray data, the expression of the gene coding for the transcription factor is considered to be an accurate surrogate for the true activity of the protein it produces. There are many instances where this is inaccurate due to post-translational modifications of the transcription factor protein. Inference of the *activity *of the transcription factor from the expression of its targets has predominantly involved linear models that do not reflect the nonlinear nature of transcription. We extend a recent approach to inferring the transcription factor activity based on nonlinear Michaelis-Menten kinetics of transcription from maximum likelihood to fully Bayesian inference and give an example of how the model can be further developed.

**Results:**

We present results on synthetic and real microarray data. Additionally, we illustrate how gene and replicate specific delays can be incorporated into the model.

**Conclusion:**

We demonstrate that full Bayesian inference is appropriate in this application and has several benefits over the maximum likelihood approach, especially when the volume of data is limited. We also show the benefits of using a non-linear model over a linear model, particularly in the case of repression.

## Background

With the increase in volume of gene expression data available from high throughput microarray experiments, much research interest has been directed at building mathematical models of the process of gene regulation. Such models have primarily been used for the so called reverse engineering of regulatory networks: inferring possible regulatory interactions and modules directly from microarray data, see for example [1-4]. All of these techniques make the implicit assumption that the expression of the transcription factor (TF) gene can be used as a proxy for the true transcription factor *activity *– the concentration of the protein in a form that is able to bind and induce/repress transcription. Whilst for some TF-gene pairs, this is likely to be a reasonable assumption, there are many examples of regulatory interactions where this is not the case due to post-transcriptional and post-translational modifications of the TF and the combinatorial effects of multiple TFs regulating a particular gene. For example, in a recent study, Newman et al [5] monitored protein levels in yeast at single-cell resolution by using novel high-throughput technology (flow cytometry and a library of green fluorescent protein-tagged yeast strains). These authors examined how protein and mRNA changes are related, in order to identify potential examples of post-transcriptional regulation. Their study identified a significant number of cases (135) where protein changes are not mirrored by mRNA changes. [5] independently verified some of these examples of post-transcriptional regulation by other quantitative techniques, such as western blotting and quantitative polymerase chain reaction. Clearly, these proteins are regulated post-transcriptionally and their changes cannot be captured on microarrays.

**Figure 1:**
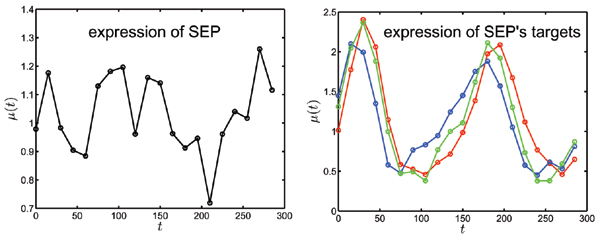
**Expression of SEP is uncorrelated with its targets**. Non-periodic expression of SEP and periodic expression of its gene targets

An example that we will use later in this paper is the TF SEP from *S. Pompbe *(fission yeast). The SEP TF regulates 15 targets, all of which are periodically expressed over the course of the cell cycle [6]. However, the expression of SEP itself is not periodically expressed as can be seen in figure 1.

To overcome the problem of the lack of correlation between the TF gene and target genes, several approaches recently proposed treat the true transcription factor activities (TFAs) as latent variables that are inferred from observed expression data of their gene-targets [7-13]. Such techniques require some prior knowledge of the network topology and are thus rather different from approaches that attempt to infer regulatory networks from microarray data alone. In applications where no topology information is available this would be a disadvantage. However, in the majority of cases some topology has been elucidated via techniques such as gene knockouts and chromatin immunoprecipitation (ChIP) assays. Moreover, *in-vitro *measurements of the levels of TFs and the rate-constants of their binding and disassociation to the promoter regions of their target genes is very difficult suggesting that inferring such quantities from the more easily obtainable expression data is a realistic and principled way forward. In this paper, we extend a recent approach for TFA inference based on a plausible, non-linear model of transcription, from a frequentist to a Bayesian framework and show the benefits of full Bayesian inference in this area with examples from both synthetic data (where the TFA is known) and real microarray data.

## Approach

To date, several approaches have been proposed to infer TFA from expression data, the majority of which have concentrated on linear models of target-gene transcription. For example, [9] use a linear model based on the technique of partial least squares (PLS) to infer TFA using regulatory topology given by chromatin immunoprecipitation (ChIP) data. A similar, probabilistic model has also recently been proposed by [7]. As well as inferring the TFA, these models have the added benefit that to some extent they can de-noise the ChIP data by removing some of the false positives. A similar method (Network Component Analysis) was previously proposed [11]. Here the authors decompose the expression matrix into a set of TFAs and weights where the decomposition is constrained to satisfy known topology. The linear assumptions simplify inference and make the algorithms useful for modeling very large data-sets. However, there are two main drawbacks to such an approach. Firstly, a realistic model of transcription should relate the rate of production of mRNA (and not its absolute value) to the TFA. Secondly, the linear model of transcription cannot encapsulate the known non-linear effects present in transcription, particularly saturation, where the rate of mRNA production reaches a natural limit due to physical constraints.

An alternative to these genome-wide approaches is to take a small subnetwork and create a more detailed, mechanistic model. Such an approach is adopted in some recent work where the transcriptional model is described using ordinary differential equations (ODEs). Firstly, [10] uses a linear ODE to define the transcriptional model for several genes that are potential targets for p53,

μ˙
 MathType@MTEF@5@5@+=feaafiart1ev1aaatCvAUfKttLearuWrP9MDH5MBPbIqV92AaeXatLxBI9gBaebbnrfifHhDYfgasaacH8akY=wiFfYdH8Gipec8Eeeu0xXdbba9frFj0=OqFfea0dXdd9vqai=hGuQ8kuc9pgc9s8qqaq=dirpe0xb9q8qiLsFr0=vr0=vr0dc8meaabaqaciaacaGaaeqabaqabeGadaaakeaaiiGacuWF8oqBgaGaaaaa@2E72@_*g*_(*t*) = *α*_*g *_+ *β*_*g*_*η*(*t*) - *δ*_*g*_*μ*_*g*_(*t*),     (1)

where *μ*_*g*_(*t*) is the expression of gene *g *at time *t*, μ˙
 MathType@MTEF@5@5@+=feaafiart1ev1aaatCvAUfKttLearuWrP9MDH5MBPbIqV92AaeXatLxBI9gBaebbnrfifHhDYfgasaacH8akY=wiFfYdH8Gipec8Eeeu0xXdbba9frFj0=OqFfea0dXdd9vqai=hGuQ8kuc9pgc9s8qqaq=dirpe0xb9q8qiLsFr0=vr0=vr0dc8meaabaqaciaacaGaaeqabaqabeGadaaakeaaiiGacuWF8oqBgaGaaaaa@2E72@_*g*_(*t*) denotes the rate of change of *μ*_*g *_at time *t *and *η*(*t*) is the TFA. Each gene is characterised by its own set of 3 kinetic parameters (*α*_*g*_, *β*_*g*_, *γ*_*g*_) and Bayesian inference is performed via Markov-chain Monte-Carlo. The three parameters all have biological interpretations; *α*_*g *_corresponds to a basal level of production, *β*_*g *_is sometimes referred to as the sensitivity and can be thought of as the level to which the production term for gene *g *is sensitive to the TFA and *δ*_*g*_*μ*_*g*_(*t*) corresponds to linear mRNA decay. Additionally, the explicit dependence on time means that the model can rigorously handle experimental readings taken at variable spacings as is often the case. The model is elegantly enhanced in [8], where it is shown that if a Gaussian process (GP) prior is placed on the TFA profile, it is possible to circumvent the need for expensive sampling-based inference and a TFA profile can be inferred over all time, rather than just at the discrete time-points at which expression was measured. These models are still limited by their linearity and the fact that they cannot properly handle repression – allowing *β *to become negative is not satisfactory as it suggests that the TF is decreasing the level of mRNA directly rather than reducing the level of production (this issue is addressed in more detail in the experimental section). In [13], the authors use a more realistic model of transcription based on the Michaelis-Menten (MM) kinetic equation, given as

Activationμ˙g(t)=αg+βgη(t)η(t)+γg−δgμg(t)Repressionμ˙g(t)=αg+βg1η(t)+γg−δgμg(t).     (2)
 MathType@MTEF@5@5@+=feaafiart1ev1aaatCvAUfKttLearuWrP9MDH5MBPbIqV92AaeXatLxBI9gBaebbnrfifHhDYfgasaacH8akY=wiFfYdH8Gipec8Eeeu0xXdbba9frFj0=OqFfea0dXdd9vqai=hGuQ8kuc9pgc9s8qqaq=dirpe0xb9q8qiLsFr0=vr0=vr0dc8meaabaqaciaacaGaaeqabaqabeGadaaakeaafaqabeGacaaabaacbaGae8xqaeKae83yamMae8hDaqNae8xAaKMae8NDayNae8xyaeMae8hDaqNae8xAaKMae83Ba8Mae8NBa4gabaacciGaf4hVd0MbaiaadaWgaaWcbaGaem4zaCgabeaakiabcIcaOiabdsha0jabcMcaPiabg2da9iab+f7aHnaaBaaaleaacqWGNbWzaeqaaOGaey4kaSIae4NSdi2aaSbaaSqaaiabdEgaNbqabaGcdaWcaaqaaiab+D7aOjabcIcaOiabdsha0jabcMcaPaqaaiab+D7aOjabcIcaOiabdsha0jabcMcaPiabgUcaRiab+n7aNnaaBaaaleaacqWGNbWzaeqaaaaakiabgkHiTiab+r7aKnaaBaaaleaacqWGNbWzaeqaaOGae4hVd02aaSbaaSqaaiabdEgaNbqabaGccqGGOaakcqWG0baDcqGGPaqkaeaacqWFsbGucqWFLbqzcqWFWbaCcqWFYbGCcqWFLbqzcqWFZbWCcqWFZbWCcqWFPbqAcqWFVbWBcqWFUbGBaeaacuGF8oqBgaGaamaaBaaaleaacqWGNbWzaeqaaOGaeiikaGIaemiDaqNaeiykaKIaeyypa0Jae4xSde2aaSbaaSqaaiabdEgaNbqabaGccqGHRaWkcqGFYoGydaWgaaWcbaGaem4zaCgabeaakmaalaaabaGaeGymaedabaGae43TdGMaeiikaGIaemiDaqNaeiykaKIaey4kaSIae43SdC2aaSbaaSqaaiabdEgaNbqabaaaaOGaeyOeI0Iae4hTdq2aaSbaaSqaaiabdEgaNbqabaGccqGF8oqBdaWgaaWcbaGaem4zaCgabeaakiabcIcaOiabdsha0jabcMcaPiabc6caUaaacaWLjaGaaCzcamaabmaabaGaeGOmaidacaGLOaGaayzkaaaaaa@960B@

Inference in [13] is performed via maximising the likelihood of the observed expression data under a log-normal noise model. Whilst the results presented look promising, there are several drawbacks in the maximum likelihood approach that are particularly acute in this application. In practice, due to the non-standard form of the model, maximising the likelihood is far from trivial. A conjugate gradient scheme is used, with multiple starting points. However, the need to calculate gradients with respect to all of the parameters being inferred imposes some constraints on the model. For example, the authors were forced to have gene-specific noise parameters, when one per dataset or replicate might be more sensible. Such constraints also severely limit the ways in which the model can be extended.

In this paper, we will show how fully Bayesian inference in the model of [13] can be performed more effectively for the following reasons. Firstly, as alluded to above, in this application it is far more straightforward to implement and much easier to extend. Secondly, it provides a principled method for the incorporation of prior biological knowledge. This may be in the form of suitable ranges for kinetic parameters, known kinetic parameter values, suitable distributions on measurement noise or known initial TFA concentrations. Thirdly, posterior distributions provide confidence in predictions – something that is particularly important when small amounts of data are available. Finally, the Bayesian approach could facilitate better experimental design. A *good *expression dataset could be considered to be one that provides the most information – a quantity that could be measured via the KL-divergence between the posterior and the prior. In addition, we will show how the model can be extended within Bayesian framework by incorporating gene and replicate specific delays.

## Methods

**Figure 2:**
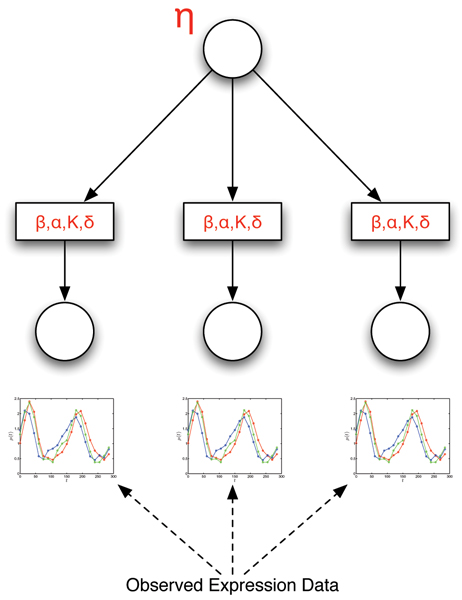
**Model description**. Cartoon depicting the model of [13]

The model of [13] is summarised in the cartoon of figure 2. The cartoon depicts the class of small regulatory subnetwork that we will be interested in here, known as a Single Input Motif (SIM) [14]. A SIM consists of one transcription factor regulating a set of *g *= 1...*G *target genes. As given in equation 2, the time derivative of the expression for a particular gene *g*, at time *t *is made up of three separate terms. A basal level of production (*α*_*g*_), a production term (varying for activation or repression, with parameters *β*_*g *_and *γ*_*g *_and depending on the TFA, *η*) and a linear decay term (with the rate parameter *δ*_*g*_). For notational convenience, we define *θ*_*g *_= {*α*_*g*_, *β*_*g*_, *γ*_*g*_, *δ*_*g*_}. The general solution of equation 2 for the case of activation is

μg(t)=(μg0−αgδg)e−δgt+αgδg+β∫0te−δ(t−τ)η(t)γg+η(t)dτ.     (3)
 MathType@MTEF@5@5@+=feaafiart1ev1aaatCvAUfKttLearuWrP9MDH5MBPbIqV92AaeXatLxBI9gBaebbnrfifHhDYfgasaacH8akY=wiFfYdH8Gipec8Eeeu0xXdbba9frFj0=OqFfea0dXdd9vqai=hGuQ8kuc9pgc9s8qqaq=dirpe0xb9q8qiLsFr0=vr0=vr0dc8meaabaqaciaacaGaaeqabaqabeGadaaakeaaiiGacqWF8oqBdaWgaaWcbaGaem4zaCgabeaakiabcIcaOiabdsha0jabcMcaPiabg2da9iabcIcaOiab=X7aTnaaBaaaleaacqWGNbWzcqaIWaamaeqaaOGaeyOeI0YaaSaaaeaacqWFXoqydaWgaaWcbaGaem4zaCgabeaaaOqaaiab=r7aKnaaBaaaleaacqWGNbWzaeqaaaaakiabcMcaPiabdwgaLnaaCaaaleqabaGaeyOeI0Iae8hTdq2aaSbaaWqaaiabdEgaNbqabaWccqWG0baDaaGccqGHRaWkdaWcaaqaaiab=f7aHnaaBaaaleaacqWGNbWzaeqaaaGcbaGae8hTdq2aaSbaaSqaaiabdEgaNbqabaaaaOGaey4kaSIae8NSdi2aa8qmaeaacqWGLbqzdaahaaWcbeqaaiabgkHiTiab=r7aKjabcIcaOiabdsha0jabgkHiTiab=r8a0jabcMcaPaaaaeaacqaIWaamaeaacqWG0baDa0Gaey4kIipakmaalaaabaGae83TdGMaeiikaGIaemiDaqNaeiykaKcabaGae83SdC2aaSbaaSqaaiabdEgaNbqabaGccqGHRaWkcqWF3oaAcqGGOaakcqWG0baDcqGGPaqkaaGaemizaqMae8hXdqNaeiOla4IaaCzcaiaaxMaadaqadaqaaiabiodaZaGaayjkaiaawMcaaaaa@75F8@

However, the expression data is observed at only a finite set of discrete timepoints, {*t*_0_,...,*t*_*i*_,...,*t*_*T*_} and so, to simplify computation and limit the number of free parameters, we make the assumption that between these time points, the TFA is constant. Hence, our inferred TFA profile will be piecewise constant.

To this end, we define η¯
 MathType@MTEF@5@5@+=feaafiart1ev1aaatCvAUfKttLearuWrP9MDH5MBPbIqV92AaeXatLxBI9gBaebbnrfifHhDYfgasaacH8akY=wiFfYdH8Gipec8Eeeu0xXdbba9frFj0=OqFfea0dXdd9vqai=hGuQ8kuc9pgc9s8qqaq=dirpe0xb9q8qiLsFr0=vr0=vr0dc8meaabaqaciaacaGaaeqabaqabeGadaaakeaaiiGacuWF3oaAgaqeaaaa@2E77@_*j *_as the constant value of TFA between *t*_*j *_and *t*_*j*+1_. With the piecewise assumption, the integral in equation 3 becomes a summation over the discrete timepoints and, defining *μ*_*gi *_as the expression of gene *g *at discrete time-point *t*_*i*_,

μgi=(μg0−αgδg)e−δti+αgδg+βge−δgti1δg∑j=0i−1(eδgtj+1− eδgtj)η¯jη¯j+γg,     (4)
 MathType@MTEF@5@5@+=feaafiart1ev1aaatCvAUfKttLearuWrP9MDH5MBPbIqV92AaeXatLxBI9gBaebbnrfifHhDYfgasaacH8akY=wiFfYdH8Gipec8Eeeu0xXdbba9frFj0=OqFfea0dXdd9vqai=hGuQ8kuc9pgc9s8qqaq=dirpe0xb9q8qiLsFr0=vr0=vr0dc8meaabaqaciaacaGaaeqabaqabeGadaaakeaafaqaaeGadaaabaacciGae8hVd02aaSbaaSqaaiabdEgaNjabdMgaPbqabaaakeaacqGH9aqpaeaacqGGOaakcqWF8oqBdaWgaaWcbaGaem4zaCMaeGimaadabeaakiabgkHiTmaalaaabaGae8xSde2aaSbaaSqaaiabdEgaNbqabaaakeaacqWF0oazdaWgaaWcbaGaem4zaCgabeaaaaGccqGGPaqkcqWGLbqzdaahaaWcbeqaaiabgkHiTiab=r7aKjabdsha0naaBaaameaacqWGPbqAaeqaaaaakiabgUcaRmaalaaabaGae8xSde2aaSbaaSqaaiabdEgaNbqabaaakeaacqWF0oazdaWgaaWcbaGaem4zaCgabeaaaaaakeaaaeaacqGHRaWkaeaacqWFYoGydaWgaaWcbaGaem4zaCgabeaakiabdwgaLnaaCaaaleqabaGaeyOeI0Iae8hTdq2aaSbaaWqaaiabdEgaNbqabaWccqWG0baDdaWgaaadbaGaemyAaKgabeaaaaGcdaWcaaqaaiabigdaXaqaaiab=r7aKnaaBaaaleaacqWGNbWzaeqaaaaakmaaqahabaGaeiikaGIaemyzau2aaWbaaSqabeaacqWF0oazdaWgaaadbaGaem4zaCgabeaaliabdsha0naaBaaameaacqWGQbGAcqGHRaWkcqaIXaqmaeqaaaaakiabgkHiTiaaykW7aSqaaiabdQgaQjabg2da9iabicdaWaqaaiabdMgaPjabgkHiTiabigdaXaqdcqGHris5aOGaemyzau2aaWbaaSqabeaacqWF0oazdaWgaaadbaGaem4zaCgabeaaliabdsha0naaBaaameaacqWGQbGAaeqaaaaakiabcMcaPmaalaaabaGaf83TdGMbaebadaWgaaWcbaGaemOAaOgabeaaaOqaaiqb=D7aOzaaraWaaSbaaSqaaiabdQgaQbqabaGccqGHRaWkcqWFZoWzdaWgaaWcbaGaem4zaCgabeaaaaGccqGGSaalaaGaaCzcaiaaxMaadaqadaqaaiabisda0aGaayjkaiaawMcaaaaa@8C66@

where *μ*_*g*0 _is the initial expression of gene *g *and will be treated as another parameter to be inferred. We must now define a noise model that will relate the predicted profiles (equation 4) to the observed expression data. Following [13], we assume that the observed expression data (on its original rather than logged scale) is log-normally distributed. The variation of variance with magnitude is a property of the log-normal distribution that is particularly desirable here. The distribution is parameterised by a location parameter *m *and a scale parameter *σ*^2^. Equating the predicted expression value from the model for gene *g *at time *t*_*i *_(*μ*_*gi*_) with the expected value of the log-normal distribution gives *m*_*gi *_= log *μ*_*gi *_- 12
 MathType@MTEF@5@5@+=feaafiart1ev1aaatCvAUfKttLearuWrP9MDH5MBPbIqV92AaeXatLxBI9gBaebbnrfifHhDYfgasaacH8akY=wiFfYdH8Gipec8Eeeu0xXdbba9frFj0=OqFfea0dXdd9vqai=hGuQ8kuc9pgc9s8qqaq=dirpe0xb9q8qiLsFr0=vr0=vr0dc8meaabaqaciaacaGaaeqabaqabeGadaaakeaadaWcaaqaaiabigdaXaqaaiabikdaYaaaaaa@2E9E@*σ*^2^. Assuming *a-priori *independence across genes, time and experimental replicates, and denoting by *x*_*gir *_the observed expression of gene *g *at time *t*_*i *_in replicate *r *(of *R *total replicates), the likelihood of the complete expression dataset **X **follows as

p(X|θ,η,σ2)=∏i=0T∏r=1R∏g=1G12πσxgirexp⁡{−(log⁡xgir−mgi)22σ2},     (5)
 MathType@MTEF@5@5@+=feaafiart1ev1aaatCvAUfKttLearuWrP9MDH5MBPbIqV92AaeXatLxBI9gBaebbnrfifHhDYfgasaacH8akY=wiFfYdH8Gipec8Eeeu0xXdbba9frFj0=OqFfea0dXdd9vqai=hGuQ8kuc9pgc9s8qqaq=dirpe0xb9q8qiLsFr0=vr0=vr0dc8meaabaqaciaacaGaaeqabaqabeGadaaakeaacqWGWbaCcqGGOaakieqacqWFybawcqGG8baFiiWacqGF4oqCcqGGSaalcqGF3oaAcqGGSaaliiGacqqFdpWCdaahaaWcbeqaaiabikdaYaaakiabcMcaPiabg2da9maarahabaWaaebCaeaadaqeWbqaamaalaaabaGaeGymaedabaWaaOaaaeaacqaIYaGmcqqFapaCaSqabaGccqqFdpWCcqWG4baEdaWgaaWcbaGaem4zaCMaemyAaKMaemOCaihabeaaaaaabaGaem4zaCMaeyypa0JaeGymaedabaGaem4raCeaniabg+GivdGccyGGLbqzcqGG4baEcqGGWbaCdaGadaqaaiabgkHiTmaalaaabaGaeiikaGIagiiBaWMaei4Ba8Maei4zaCMaemiEaG3aaSbaaSqaaiabdEgaNjabdMgaPjabdkhaYbqabaGccqGHsislcqWGTbqBdaWgaaWcbaGaem4zaCMaemyAaKgabeaakiabcMcaPmaaCaaaleqabaGaeGOmaidaaaGcbaGaeGOmaiJae03Wdm3aaWbaaSqabeaacqaIYaGmaaaaaaGccaGL7bGaayzFaaGaeiilaWIaaCzcaiaaxMaadaqadaqaaiabiwda1aGaayjkaiaawMcaaaWcbaGaemOCaiNaeyypa0JaeGymaedabaGaemOuaifaniabg+GivdaaleaacqWGPbqAcqGH9aqpcqaIWaamaeaacqWGubava0Gaey4dIunaaaa@7CD0@

where we have added *μ*_*g*0 _to the set of parameters *θ*_*g *_for each gene and defined ***θ ***= {*θ*_1_,...,*θ*_*G*_} and ***η ***= {η¯
 MathType@MTEF@5@5@+=feaafiart1ev1aaatCvAUfKttLearuWrP9MDH5MBPbIqV92AaeXatLxBI9gBaebbnrfifHhDYfgasaacH8akY=wiFfYdH8Gipec8Eeeu0xXdbba9frFj0=OqFfea0dXdd9vqai=hGuQ8kuc9pgc9s8qqaq=dirpe0xb9q8qiLsFr0=vr0=vr0dc8meaabaqaciaacaGaaeqabaqabeGadaaakeaaiiGacuWF3oaAgaqeaaaa@2E77@_0_,...,η¯
 MathType@MTEF@5@5@+=feaafiart1ev1aaatCvAUfKttLearuWrP9MDH5MBPbIqV92AaeXatLxBI9gBaebbnrfifHhDYfgasaacH8akY=wiFfYdH8Gipec8Eeeu0xXdbba9frFj0=OqFfea0dXdd9vqai=hGuQ8kuc9pgc9s8qqaq=dirpe0xb9q8qiLsFr0=vr0=vr0dc8meaabaqaciaacaGaaeqabaqabeGadaaakeaaiiGacuWF3oaAgaqeaaaa@2E77@_*T*-1_}. The scale parameter of the log-normal distribution, denoted *σ*^2 ^is treated as an additional model parameter to be inferred. Note that unlike in [13], we are free to use one noise parameter for the whole dataset or index it with genes or replicates as desired.

To ensure all of the kinetic parameters remain positive, we will take the standard step of sampling in their log space, and place uniform priors (between 0 and 30) over their values to encapsulate our lack of prior information. Additionally, we place a uniform prior (between 0 and 10) on η¯
 MathType@MTEF@5@5@+=feaafiart1ev1aaatCvAUfKttLearuWrP9MDH5MBPbIqV92AaeXatLxBI9gBaebbnrfifHhDYfgasaacH8akY=wiFfYdH8Gipec8Eeeu0xXdbba9frFj0=OqFfea0dXdd9vqai=hGuQ8kuc9pgc9s8qqaq=dirpe0xb9q8qiLsFr0=vr0=vr0dc8meaabaqaciaacaGaaeqabaqabeGadaaakeaaiiGacuWF3oaAgaqeaaaa@2E77@ and a Gamma prior on *σ*^2 ^(with parameters *a *= 0.1, *b *= 1, such that the expected value of *σ*^2 ^under the prior is 0.1). Finally, inspection of equation 4 shows that there is a coupling between *η *and *γ*_*g*_. In the synthetic experiments, we are interested in comparing inferred parameter values with the known values and so we overcome this problem by fixing η¯
 MathType@MTEF@5@5@+=feaafiart1ev1aaatCvAUfKttLearuWrP9MDH5MBPbIqV92AaeXatLxBI9gBaebbnrfifHhDYfgasaacH8akY=wiFfYdH8Gipec8Eeeu0xXdbba9frFj0=OqFfea0dXdd9vqai=hGuQ8kuc9pgc9s8qqaq=dirpe0xb9q8qiLsFr0=vr0=vr0dc8meaabaqaciaacaGaaeqabaqabeGadaaakeaaiiGacuWF3oaAgaqeaaaa@2E77@_0 _to the true value. In experiments with real data, the problem of arbitrary re-scaling is effectively side-stepped by the restrictions imposed on ***η ***through its uniform prior.

Using **Δ **to denote the set of hyper-parameters used to define the various priors, the full posterior over ***θ***, ***η ***and *σ*^2 ^is given by

p(η,θ,σ2|X,Δ)=p(X|θ,η,σ2)p(θ,η,σ2|Δ)∫p(X|θ,η,σ2)p(θ,η,σ2|Δ)dθdηdσ2.     (6)
 MathType@MTEF@5@5@+=feaafiart1ev1aaatCvAUfKttLearuWrP9MDH5MBPbIqV92AaeXatLxBI9gBaebbnrfifHhDYfgasaacH8akY=wiFfYdH8Gipec8Eeeu0xXdbba9frFj0=OqFfea0dXdd9vqai=hGuQ8kuc9pgc9s8qqaq=dirpe0xb9q8qiLsFr0=vr0=vr0dc8meaabaqaciaacaGaaeqabaqabeGadaaakeaacqWGWbaCcqGGOaakiiWacqWF3oaAcqGGSaalcqWF4oqCcqGGSaaliiGacqGFdpWCdaahaaWcbeqaaiabikdaYaaakiabcYha8Hqabiab9HfayjabcYcaSGGabiab8r5aejabcMcaPiabg2da9maalaaabaGaemiCaaNaeiikaGIae0hwaGLaeiiFaWNae8hUdeNaeiilaWIae83TdGMaeiilaWIae43Wdm3aaWbaaSqabeaacqaIYaGmaaGccqGGPaqkcqWGWbaCcqGGOaakcqWF4oqCcqGGSaalcqWF3oaAcqGGSaalcqGFdpWCdaahaaWcbeqaaiabikdaYaaakiabcYha8jab8r5aejabcMcaPaqaamaapeaabaGaemiCaaNaeiikaGIae0hwaGLaeiiFaWNae8hUdeNaeiilaWIae83TdGMaeiilaWIae43Wdm3aaWbaaSqabeaacqaIYaGmaaGccqGGPaqkcqWGWbaCcqGGOaakcqWF4oqCcqGGSaalcqWF3oaAcqGGSaalcqGFdpWCdaahaaWcbeqaaiabikdaYaaakiabcYha8jab8r5aejabcMcaPiabdsgaKjab=H7aXjabdsgaKjab=D7aOjabdsgaKjab+n8aZnaaCaaaleqabaGaeGOmaidaaaqabeqaniabgUIiYdaaaOGaeiOla4IaaCzcaiaaxMaadaqadaqaaiabiAda2aGaayjkaiaawMcaaaaa@8649@

To obtain samples from this posterior, we use the well-known Metropolis algorithm (see, for example, [15]). For each of our parameters, we use a Gaussian proposal distribution. An initial number of samples is used to estimate the variance of the proposal distribution. This is then tuned during the burn-in period to try and achieve an efficient acceptance rate between 20–40% as suggested in [15]. Convergence is assessed by running 10 separate chains and monitoring the within and between chain variance of each parameter (see for example [15], p.296). The sampler is assumed to have converged when the value of R^
 MathType@MTEF@5@5@+=feaafiart1ev1aaatCvAUfKttLearuWrP9MDH5MBPbIqV92AaeXatLxBI9gBaebbnrfifHhDYfgasaacH8akY=wiFfYdH8Gipec8Eeeu0xXdbba9frFj0=OqFfea0dXdd9vqai=hGuQ8kuc9pgc9s8qqaq=dirpe0xb9q8qiLsFr0=vr0=vr0dc8meaabaqaciaacaGaaeqabaqabeGadaaakeaacuWGsbGugaqcaaaa@2DE9@ (see [15], p.297) for every parameter is below 1.1.

## Results and discussion

### Synthetic data example

**Figure 3:**
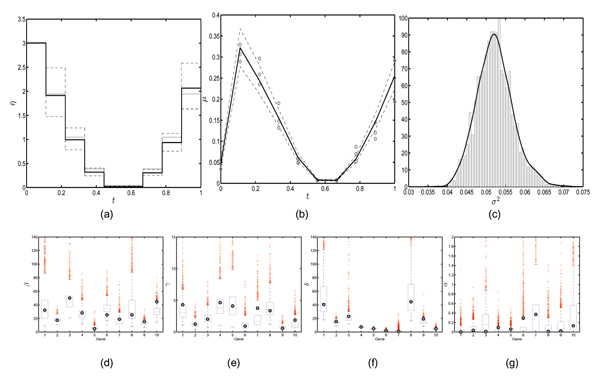
**Synthetic Example**. Synthetic data example. (a) shows the true and inferred η¯
 MathType@MTEF@5@5@+=feaafiart1ev1aaatCvAUfKttLearuWrP9MDH5MBPbIqV92AaeXatLxBI9gBaebbnrfifHhDYfgasaacH8akY=wiFfYdH8Gipec8Eeeu0xXdbba9frFj0=OqFfea0dXdd9vqai=hGuQ8kuc9pgc9s8qqaq=dirpe0xb9q8qiLsFr0=vr0=vr0dc8meaabaqaciaacaGaaeqabaqabeGadaaakeaaiiGacuWF3oaAgaqeaaaa@2E77@ profiles (note, in all figures, dashed lines correspond to the 5th and 95th percentiles). (b) Expression data and inferred profile for a typical gene. (c) Posterior for *σ*^2^, true value was 0.05. (d)–(g) Posteriors for kinetic paramaters, *β*, *γ*, *δ*, *α *respectively. The boxes represent the region between the 25th and 75th percentiles with the median shown. Dotted lines give the range of the data, with outliers shown as crosses. Gray circles correspond to the true values.

**Figure 4:**
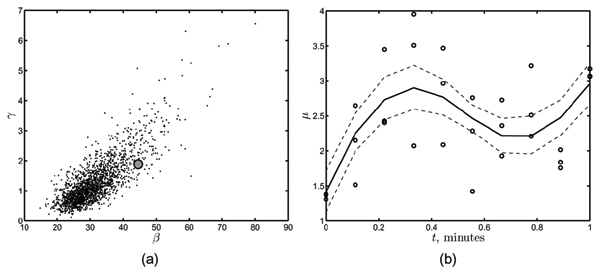
**Synthetic Example – problem genes**. Dependency between *β *and *γ *and high noise in data for two genes in the synthetic example. (a) Posterior samples for *β *and *γ *for gene 10 in the synthetic example, (b) *μ *for gene 7 and expression data. The high level of noise leads to the poor parameter inference in this case.

Consider a SIM consisting of 10 target genes all activated by the same TF. Using the true *η *profile shown in figure 3a, three expression data-sets were synthesised according to MM kinetics (equation 4) with *σ*^2 ^= 0.05 and three replicates. From figure 3a, it is clear that the inferred mean *η *profile closely corresponds to the true profile from which the data was produced. Figure 3b shows the 3 replicates generated for one particular gene as well as the mean and percentiles of *μ *and the inferred density over *σ*^2 ^is highly concentrated about the true value and shown in figure 3c. Figures 3d to 3g show samples from the posteriors for the four kinetic parameters over the ten genes as box plots. We notice that in the majority of the genes, the posteriors for *β*, *γ *and *δ *are concentrated around the true values. The distributions for *α *however, are rather less convincing but given the scale of *α *relative to the other parameters (particularly *β*), the deviations from the true values are relatively insignificant. Two genes, numbers 7 and 10, seem to have posterior *β *and *γ *distributions that have only very low mass at the true values. Examining the posterior samples for *β *and *γ *for gene 10 shown in figure 4a, it is clear that the two parameters are dependent and the ratio of *β *to *γ *at the mode is very close to the ratio at the true value. The fit to the expression data is also very good (not shown). The ability to visualise the posteriors in this way is a clear advantage over the maximum likelihood framework where obtaining asymptotic approximations of the covariance from second derivatives of the likelihood is not at all straightforward and are also likely to be inaccurate with small amounts of data. The inferred *μ *profile for gene 7 on the other hand does not fit the observed expression data well (see figure 4b) due to the large relative magnitude of the noise in the observations and in this case it is unsurprising that the inference is cautious – indicated by the width of the posteriors. Despite these data problems, the inferred TFA profile in which we are ultimately interested is very close to the true profile.

### Fission yeast cell cycle data

The reconstruction approach is now applied to the cell-cycle microarray data of *S. pombe *or fission yeast [6]. This dataset contains two time-course experiments obtained using different cell-cycle synchronization methods. One method is centrifugal elutriation, which generates a homogeneous population of small cells early in their cell cycle. There are 3 independent biological replicates available, each contains 20 time-points, taken every 15 minutes and it is this data that we will use here. Elsewhere [16], we have shown how the MM framework can be extended to combine the data from elutration synchronisation with the data from the alternative synchronisation which has samples at different time points.

[6] study three transcription factors that are involved in regulating three different groups of genes in the fission yeast cell-cycle (see also [17]) and we will restrict ourselves to one SIM, regulated by a transcription factor complex, known to involve SEP. The 14 targets are taken from experiments of Rustici et al (2004) (see their Figure 3). In addition, gene ace2, which codes for Ace2p, is known to be the target of Sep1p [17]. It is included in the SIM as another target. Imputing of the missing values in the data has been done using impute.missing() function from smida [18].

**Figure 5:**
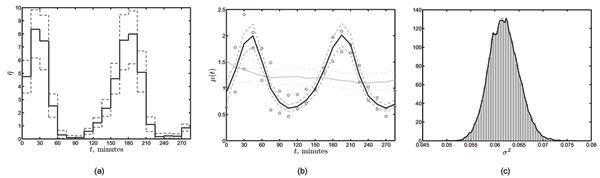
**Fission yeast example**. Example of inference with real microarray data from fission yeast. (a), Inferred mean η¯
 MathType@MTEF@5@5@+=feaafiart1ev1aaatCvAUfKttLearuWrP9MDH5MBPbIqV92AaeXatLxBI9gBaebbnrfifHhDYfgasaacH8akY=wiFfYdH8Gipec8Eeeu0xXdbba9frFj0=OqFfea0dXdd9vqai=hGuQ8kuc9pgc9s8qqaq=dirpe0xb9q8qiLsFr0=vr0=vr0dc8meaabaqaciaacaGaaeqabaqabeGadaaakeaaiiGacuWF3oaAgaqeaaaa@2E77@ profile with 5th and 95th percentiles, (b) *μ *for gene 1 when η¯
 MathType@MTEF@5@5@+=feaafiart1ev1aaatCvAUfKttLearuWrP9MDH5MBPbIqV92AaeXatLxBI9gBaebbnrfifHhDYfgasaacH8akY=wiFfYdH8Gipec8Eeeu0xXdbba9frFj0=OqFfea0dXdd9vqai=hGuQ8kuc9pgc9s8qqaq=dirpe0xb9q8qiLsFr0=vr0=vr0dc8meaabaqaciaacaGaaeqabaqabeGadaaakeaaiiGacuWF3oaAgaqeaaaa@2E77@ is inferred (black lines) and when it is fixed (gray lines), (c) Posterior distribution for *σ*^2^.

In a few cases, where more than 50% missing values for a particular gene replicate, those were substituted by the means of the remaining ones. Additionally, there are rare cases where the complete data for a gene in one replicate is missing. In these cases, we replaced the data for the missing replicate with data from one of the other replicates (see, for example figure 5b). Figure 5a shows the results of performing inference on this data. The inferred TFA profile can be seen in figure 5a. We can see from the percentiles that the profile is reasonably well defined. In figure 5b we can see the expression data for one particular gene along with the mean and 5th and 95th percentiles of *μ *defined by the model (black solid and dashed lines). Finally, figure 5c shows the posterior for *σ*^2^. Note that the level of noise used to sample data in the previous section is similar to that found in this real data.

As one might expect, the TFA is periodic with two clear cycles. Interestingly, in the second cycle, the TFA seems to rise much more slowly than it drops. Such information may help to unravel the cause of the very low correlation between the SEP gene and its targets. By way of comparison, in figure 5b we have also shown the inferred *μ *profile for the same gene when the TFA is fixed at the expression of the SEP gene (shown in figure 1). It is clear that with the TFA fixed at the expression of SEP, the model is unable to explain the observed data. If the model could explain the observed data, it might suggest that the MM model is too flexible. It also provides evidence to suggest that there are indeed unobservable modifications of SEP and perhaps additional regulators in the complex.

### Incorporating delays

One major advantage of the fully Bayesian framework in this application is that it is straightforward to extend the model. One example is the integration of different data-sets that we have presented elsewhere [16]. Another example of this is the incorporation of time delays that inevitably occur between TF binding to the promoter and gene transcription. Although the various genes are all regulated by the same TF, it is clear from the data that some react quicker than others, possibly due to different promoter binding efficiencies or faster transcription rates. Due to the piecewise constant assumption on the TFA profile, we calculate effective η¯
 MathType@MTEF@5@5@+=feaafiart1ev1aaatCvAUfKttLearuWrP9MDH5MBPbIqV92AaeXatLxBI9gBaebbnrfifHhDYfgasaacH8akY=wiFfYdH8Gipec8Eeeu0xXdbba9frFj0=OqFfea0dXdd9vqai=hGuQ8kuc9pgc9s8qqaq=dirpe0xb9q8qiLsFr0=vr0=vr0dc8meaabaqaciaacaGaaeqabaqabeGadaaakeaaiiGacuWF3oaAgaqeaaaa@2E77@ values as linear combinations of consecutive η¯
 MathType@MTEF@5@5@+=feaafiart1ev1aaatCvAUfKttLearuWrP9MDH5MBPbIqV92AaeXatLxBI9gBaebbnrfifHhDYfgasaacH8akY=wiFfYdH8Gipec8Eeeu0xXdbba9frFj0=OqFfea0dXdd9vqai=hGuQ8kuc9pgc9s8qqaq=dirpe0xb9q8qiLsFr0=vr0=vr0dc8meaabaqaciaacaGaaeqabaqabeGadaaakeaaiiGacuWF3oaAgaqeaaaa@2E77@ values. Denoting by *τ*_*g *_the delay for gene *g*, a delay of 0.2 time steps suggests that the effective TFA (η^
 MathType@MTEF@5@5@+=feaafiart1ev1aaatCvAUfKttLearuWrP9MDH5MBPbIqV92AaeXatLxBI9gBaebbnrfifHhDYfgasaacH8akY=wiFfYdH8Gipec8Eeeu0xXdbba9frFj0=OqFfea0dXdd9vqai=hGuQ8kuc9pgc9s8qqaq=dirpe0xb9q8qiLsFr0=vr0=vr0dc8meaabaqaciaacaGaaeqabaqabeGadaaakeaaiiGacuWF3oaAgaqcaaaa@2E6F@_*i*_) should be 0.8η¯
 MathType@MTEF@5@5@+=feaafiart1ev1aaatCvAUfKttLearuWrP9MDH5MBPbIqV92AaeXatLxBI9gBaebbnrfifHhDYfgasaacH8akY=wiFfYdH8Gipec8Eeeu0xXdbba9frFj0=OqFfea0dXdd9vqai=hGuQ8kuc9pgc9s8qqaq=dirpe0xb9q8qiLsFr0=vr0=vr0dc8meaabaqaciaacaGaaeqabaqabeGadaaakeaaiiGacuWF3oaAgaqeaaaa@2E77@_*i *_+ 0.2η¯
 MathType@MTEF@5@5@+=feaafiart1ev1aaatCvAUfKttLearuWrP9MDH5MBPbIqV92AaeXatLxBI9gBaebbnrfifHhDYfgasaacH8akY=wiFfYdH8Gipec8Eeeu0xXdbba9frFj0=OqFfea0dXdd9vqai=hGuQ8kuc9pgc9s8qqaq=dirpe0xb9q8qiLsFr0=vr0=vr0dc8meaabaqaciaacaGaaeqabaqabeGadaaakeaaiiGacuWF3oaAgaqeaaaa@2E77@_*i*-1_. We must now also define the value of η¯
 MathType@MTEF@5@5@+=feaafiart1ev1aaatCvAUfKttLearuWrP9MDH5MBPbIqV92AaeXatLxBI9gBaebbnrfifHhDYfgasaacH8akY=wiFfYdH8Gipec8Eeeu0xXdbba9frFj0=OqFfea0dXdd9vqai=hGuQ8kuc9pgc9s8qqaq=dirpe0xb9q8qiLsFr0=vr0=vr0dc8meaabaqaciaacaGaaeqabaqabeGadaaakeaaiiGacuWF3oaAgaqeaaaa@2E77@ before *t *= 0. In the absence of any further information, we assume that η¯
 MathType@MTEF@5@5@+=feaafiart1ev1aaatCvAUfKttLearuWrP9MDH5MBPbIqV92AaeXatLxBI9gBaebbnrfifHhDYfgasaacH8akY=wiFfYdH8Gipec8Eeeu0xXdbba9frFj0=OqFfea0dXdd9vqai=hGuQ8kuc9pgc9s8qqaq=dirpe0xb9q8qiLsFr0=vr0=vr0dc8meaabaqaciaacaGaaeqabaqabeGadaaakeaaiiGacuWF3oaAgaqeaaaa@2E77@_*i *_= η¯
 MathType@MTEF@5@5@+=feaafiart1ev1aaatCvAUfKttLearuWrP9MDH5MBPbIqV92AaeXatLxBI9gBaebbnrfifHhDYfgasaacH8akY=wiFfYdH8Gipec8Eeeu0xXdbba9frFj0=OqFfea0dXdd9vqai=hGuQ8kuc9pgc9s8qqaq=dirpe0xb9q8qiLsFr0=vr0=vr0dc8meaabaqaciaacaGaaeqabaqabeGadaaakeaaiiGacuWF3oaAgaqeaaaa@2E77@_0_, ∀*i *< 0. In addition to gene specific delays, inspection of the expression data also suggests that there are replicate dependent shifts too, most likely due to the imperfect nature of the cell synchronisation procedure. Such discrepencies beg the question of whether or not one can really use the replicates together in a straightforward manner or whether the replicates need to be intelligently calibrated in some manner prior to analysis. Recently, [19] presented a method for fusing together replicates based on linear regression. Indeed, the ability to reliably combine several data-sets together is highly desirable as one large dataset is potentially more useful than several smaller ones. Hence, we introduce two additional parameters for each replicate, *ρ*_*r *_and a replicate specific noise parameter σr2
 MathType@MTEF@5@5@+=feaafiart1ev1aaatCvAUfKttLearuWrP9MDH5MBPbIqV92AaeXatLxBI9gBaebbnrfifHhDYfgasaacH8akY=wiFfYdH8Gipec8Eeeu0xXdbba9frFj0=OqFfea0dXdd9vqai=hGuQ8kuc9pgc9s8qqaq=dirpe0xb9q8qiLsFr0=vr0=vr0dc8meaabaqaciaacaGaaeqabaqabeGadaaakeaaiiGacqWFdpWCdaqhaaWcbaGaemOCaihabaGaeGOmaidaaaaa@3102@. In order to ensure that the gene delays and replicate shifts are identifiable it is necessary to fix at least one *τ*_*g *_and one *ρ*_*r *_and define all delays relative to them. Finally, we must define a prior distribution over *τ *and *ρ*. For convenience, we will fix *τ*_*g *_= 0 for the 'fastest' gene and *ρ*_*r *_= 0 for the 'fastest' replicate thus constraining all other values to be positive. For all delays and replicate shifts we use a Gamma prior with parameters *a *= 0.5, *b *= 1.

**Figure 6:**
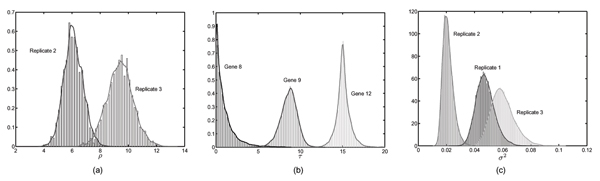
**Adding delays**. Posteriors from the delay example. (a) shows the posterior distributions for replicates 2 and 3 (note that *ρ*_1 _has been fixed at 0). (b) shows the distribution of *τ *for three particular genes. (c) shows the difference in noise levels for the three replicates.

Figure 6a gives the posterior over *ρ *(recall that *ρ*_1 _is set to 0), we can see that there is quite a significant time difference between all three replicates suggesting that some alignment of replicates would be desirable before further analysis. Being able to infer such shifts accurately for areas of the network where topology is known will undoubtedly facilitate topology inference in other areas. Secondly, figure 6b shows the posterior delay distributions for three genes. Gene 8 is typical of the majority of gene-targets and has a very small delay (relative to the fastest – gene 2). Genes 9 and 12 on the other hand, appear to have very significant delays, with 12 having a delay of the order of 15 minutes, equivalent to the sample time for the expression data. Such information is potentially useful, for example, if the delays are due to the efficiency of TF binding to the promoter then a ranking of the genes may help improve binding site discovery.

Alternatively, varying delays could be due to the different functional roles of genes (as shown, for example, in [20] for *E. coli*). In addition, many regulatory modules form cascades with genes regulated at one level also regulating their gene-targets. Standard knock-out experiments will highlight all genes downstream of a particular TF but will not be able to distinguish between direct and indirect relationships. As genes further downstream will have larger delays, being able to accurately calculate these may help in uncovering the true network structure (work in progress). Additionally, taking into account such delays will also improve the accuracy of the inferred TFA profile. Finally, it is interesting to look at the posteriors over the noise parameters, shown in figure 6c indicating a large variation in the level of noise across the three replicates.

### Is the non-linear model necessary?

**Figure 7:**
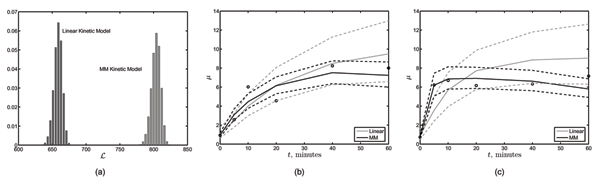
**Linear versus non-linear**. Benefits of the nonlinear model. (a) shows the significant improvement in likelihood over a linear model for the fission data. (b) a gene from the *E. coli *dataset that is modeled reasonably well by the linear model. (c) a gene from the *E. coli *dataset that is modeled badly by the linear model.

In the previous section, we saw how the TFA inference procedure can be applied to a cell-cycle regulated motif from fission yeast. However, the problem appears rather straightforward and it could be argued that a linear model could perform the same analysis. Consider the following linear ODE,

μ˙
 MathType@MTEF@5@5@+=feaafiart1ev1aaatCvAUfKttLearuWrP9MDH5MBPbIqV92AaeXatLxBI9gBaebbnrfifHhDYfgasaacH8akY=wiFfYdH8Gipec8Eeeu0xXdbba9frFj0=OqFfea0dXdd9vqai=hGuQ8kuc9pgc9s8qqaq=dirpe0xb9q8qiLsFr0=vr0=vr0dc8meaabaqaciaacaGaaeqabaqabeGadaaakeaaiiGacuWF8oqBgaGaaaaa@2E72@_*g*_(*t*) = *β*_*g*_*η*(*t*) - *δ*_*g*_*μ*_*g*_(*t*)     (7)

where we have removed the saturation (similar to [10]) and basal production parameters (these were all very close to zero in this data). Results obtained with this model, are qualitatively similar to those obtained previously with MM. However, we can objectively compare the two models using approximate Bayes' factors calculated from the following approximation of the marginal likelihood Ns/∑s=1Nsp(X|η¯s,θs,σs2)−1
 MathType@MTEF@5@5@+=feaafiart1ev1aaatCvAUfKttLearuWrP9MDH5MBPbIqV92AaeXatLxBI9gBaebbnrfifHhDYfgasaacH8akY=wiFfYdH8Gipec8Eeeu0xXdbba9frFj0=OqFfea0dXdd9vqai=hGuQ8kuc9pgc9s8qqaq=dirpe0xb9q8qiLsFr0=vr0=vr0dc8meaabaqaciaacaGaaeqabaqabeGadaaakeaacqWGobGtdaWgaaWcbaGaem4Camhabeaakiabc+caVmaaqadabaGaemiCaaNaeiikaGccbeGae8hwaGLaeiiFaWhcciGaf43TdGMbaebadaWgaaWcbaGaem4CamhabeaakiabcYcaSGGadiab9H7aXnaaBaaaleaacqWGZbWCaeqaaOGaeiilaWIae43Wdm3aa0baaSqaaiabdohaZbqaaiabikdaYaaakiabcMcaPmaaCaaaleqabaGaeyOeI0IaeGymaedaaaqaaiabdohaZjabg2da9iabigdaXaqaaiabd6eaonaaBaaameaacqWGZbWCaeqaaaqdcqGHris5aaaa@4D42@ where *N*_*s *_is the number of samples drawn and η¯
 MathType@MTEF@5@5@+=feaafiart1ev1aaatCvAUfKttLearuWrP9MDH5MBPbIqV92AaeXatLxBI9gBaebbnrfifHhDYfgasaacH8akY=wiFfYdH8Gipec8Eeeu0xXdbba9frFj0=OqFfea0dXdd9vqai=hGuQ8kuc9pgc9s8qqaq=dirpe0xb9q8qiLsFr0=vr0=vr0dc8meaabaqaciaacaGaaeqabaqabeGadaaakeaaiiGacuWF3oaAgaqeaaaa@2E77@_*s*_, ***θ***_*s*_, σs2
 MathType@MTEF@5@5@+=feaafiart1ev1aaatCvAUfKttLearuWrP9MDH5MBPbIqV92AaeXatLxBI9gBaebbnrfifHhDYfgasaacH8akY=wiFfYdH8Gipec8Eeeu0xXdbba9frFj0=OqFfea0dXdd9vqai=hGuQ8kuc9pgc9s8qqaq=dirpe0xb9q8qiLsFr0=vr0=vr0dc8meaabaqaciaacaGaaeqabaqabeGadaaakeaaiiGacqWFdpWCdaqhaaWcbaGaem4CamhabaGaeGOmaidaaaaa@3104@ are the parameter values of the *s*th draw from the posterior. The Bayes' factor (*B*) is given by the ratio of the marginal likelihood under the two competing models (assuming no *a priori *preference for either). Taking posterior samples for both the MM and linear models, we find that the 2 log *B *= 289 suggesting a great deal of evidence for the MM model ([21] suggests that 2 log *B *> 10 provides very strong evidence). The approximation to the marginal likelihood that we have used is known to have its faults (see eg [21]) and so we also adopt the ratio of likelihoods test that allows us to compare likelihoods whilst penalising the added complexity of the MM model. In figure 7a we show histograms of the log-likelihood values of the samples drawn from the posterior under the linear and MM models. Using the ratio of likelihoods test with 30 degrees of freedom (equivalent to the additional 2 parameters per gene in the MM model) the log likelihoods would have to differ by approximately 25.4 to give a significant improvement at the 1% level. It is clear from the figure that this is easily the case. In a more general model comparison scenario (i.e. comparing alternative topologies), the difference between two models is unlikely to be so extreme and so investigating more reliable approximations to the marginal likelihood is an area of ongoing investigation. As a second example, we consider a dataset for *E. coli *(from [22]) that highlights the need for a nonlinear model when the TF acts as a repressor. The linear model defined by equation 1 is used with *β *constrained to be negative. We have already discussed how this particular model is not very biologically interpretable, however it could be argued that this is not terribly important if it can adequately describe the data. One interesting characteristic of this data is that the expression profiles of the target genes are rather uncorrelated and appear to fall into two characteristic groups. Figures 7b and 7c show examples of genes from both of these groups. In 7b we see that the expression profile rises gradually throughout the time course and whilst the MM model fits the data better, the linear model captures the general trend reasonably well. However, in 7c the expression profile rises rapidly to a steady value. In this case, the linear model fails to adequately model the observed data whilst the MM model is able to describe this behavior due to the inclusion of a saturation term. Additionally, in this example, 2 log *B *= 137 (where *B *is the ratio of the marginal likelihood under the MM model to the marginal likelihood under a linear model) which again suggests strong evidence in favour of the MM model. This example highlights the necessity of a nonlinear model in this particular application as genes regulated by the same TF can have uncorrelated behavior that cannot be handled by linear models. This is a particularly acute issue when a TFA is used to suggest possible new target genes (as investigated in [10]) as essentially only candidate genes that are correlated with current known targets will be suggested.

In addition, the low quantity of data in this example shows the advantage of the Bayesian approach. The percentiles in figures 7b and 7c are rather wide, as are the posteriors for the kinetic parameters and the η¯
 MathType@MTEF@5@5@+=feaafiart1ev1aaatCvAUfKttLearuWrP9MDH5MBPbIqV92AaeXatLxBI9gBaebbnrfifHhDYfgasaacH8akY=wiFfYdH8Gipec8Eeeu0xXdbba9frFj0=OqFfea0dXdd9vqai=hGuQ8kuc9pgc9s8qqaq=dirpe0xb9q8qiLsFr0=vr0=vr0dc8meaabaqaciaacaGaaeqabaqabeGadaaakeaaiiGacuWF3oaAgaqeaaaa@2E77@ profile (not shown) providing an objective indication of the certainty/uncertainty in our predictions. Without such knowledge, it may not be clear whether the MM or linear models is more suited to the problem. However, the percentiles shown provide evidence that the nonlinear model is better supported. Finally, the MM model is not the only nonlinear model that could be used. For example, replacing η¯
 MathType@MTEF@5@5@+=feaafiart1ev1aaatCvAUfKttLearuWrP9MDH5MBPbIqV92AaeXatLxBI9gBaebbnrfifHhDYfgasaacH8akY=wiFfYdH8Gipec8Eeeu0xXdbba9frFj0=OqFfea0dXdd9vqai=hGuQ8kuc9pgc9s8qqaq=dirpe0xb9q8qiLsFr0=vr0=vr0dc8meaabaqaciaacaGaaeqabaqabeGadaaakeaaiiGacuWF3oaAgaqeaaaa@2E77@ in equation 1 with its reciprocal may also work adequately. However, inspection of the posteriors over *γ*_*g *_(not shown) shows some variation, suggesting varying saturation effects between the genes. A comparison between different nonlinear models is an avenue for future work, although the diffuse nature of the posteriors over *γ *here suggest a larger dataset would be required to come to any definite conclusions.

## Conclusion

In this paper, we have presented a fully Bayesian approach for the inference of TFA from the expression of target genes. There are many known cases (and likely many unknown) where the expression of the gene coding for the TF is highly un-correlated with the expression of its targets. In such situations, the expression of the TF cannot be used to directly model the expression of the target genes and inference of the TFA from microarray data is less expensive and more straightforward than *in-vitro *measurements. Previous approaches to TFA inference have generally assumed linear or log-linear models of transcription. However, the non-linear approach here is able to handle effects such as saturation that are known to be a part of transcription and can adequately handle both activation and repression. In addition, the MM kinetic model does not require evenly spaced expression data and modeling the rate of mRNA production rather than the absolute magnitude is more biologically plausible. We have highlighted the drawbacks of the linear model with a repression example from *E. coli*. The linear model was unable to capture the diversity in expression profiles present in one SIM. In addition, using the linear model in the repression case requires a *negative *production term that is not particularly biologically plausible. Originally, [13] proposed a maximum likelihood scheme for inferring the TFA profile. However, due to the form of the MM kinetic model, maximisation of the likelihood is not straightforward and hence here we have adopted a full Bayesian sampling strategy. As well as being more amenable to this particular application, the fully Bayesian scheme offers several other advantages, particularly that the full posterior distribution provides far more information than the maximum likelihood estimate. The shape of the posterior distributions provides information on the certainty that can be placed on subsequent inferences made.

In addition, as an example of how the model could be extended, we have shown how incorporation of delays – both biological delays specific to genes and replicate specific delays that appear as artifacts of the experimental procedure – can be accomodated within this framework. The results obtained from this analysis open some interesting questions. For example, is it sufficient to use replicates as they are provided or do they need some kind of alignment beforehand? Our results suggested that there were lags between replicates of the order of half a time interval and hence assuming that all measurements were taken at the same point in the cell cycle could be rather misleading. Values for replicate shifts inferred with this method could be used to align data for other genes that belong to areas of the regulatory network where topology is partly or totally unknown, making the data more reliable. The method also allows the disambiguation of observed time lags that are due to experimental artifacts such as shifts between replicates and genuine biological effects like different delays between genes. One area of future work that may improve the inference of delays is the investigation of richer, informative priors for *η*(*t*). A Gaussian process prior (as suggested in [8]) would allow the TFA to be defined at all time points and also provides a means for encoding a desirable *a-priori *preference for smooth functions.

## Authors' contributions

SR and RK contributed equally to this work. All authors worked on and have approved the final manuscript.
